# Persistent *Salmonella enterica* serovar Typhi sub-populations within host interrogated by whole genome sequencing and metagenomics

**DOI:** 10.1371/journal.pone.0289070

**Published:** 2023-08-23

**Authors:** Eby M. Sim, Qinning Wang, Peter Howard, Rady Kim, Ling Lim, Kirsty Hope, Vitali Sintchenko

**Affiliations:** 1 Sydney Institute for Infectious Diseases, The University of Sydney, Westmead, New South Wales, Australia; 2 Institute of Clinical Pathology and Medical Research, NSW Health Pathology, Westmead, New South Wales, Australia; 3 Centre for Infectious Diseases and Microbiology- Public Health, Westmead Hospital, Westmead, New South Wales, Australia; 4 Health Protection, New South Wales Ministry of Health, North Sydney, New South Wales, Australia; University of Helsinki: Helsingin Yliopisto, FINLAND

## Abstract

*Salmonella enterica* serovar Typhi (*S*. Typhi) causes typhoid fever and, in some cases, chronic carriage after resolution of acute disease. This study examined sequential isolates of *S*. Typhi from a single host with persistent asymptomatic infection. These isolates, along with another *S*. Typhi isolate recovered from a household contact with typhoid fever, were subjected to whole genome sequencing and analysis. In addition, direct sequencing of the bile fluid from the host with persistent infection was also performed. Comparative analysis of isolates revealed three sub-populations of *S*. Typhi with distinct genetic patterns. Metagenomic sequencing recognised only two of the three sub-populations within the bile fluid. The detection and investigation of insertion sequences IS*10R* and associated deletions complemented analysis of single nucleotide polymorphisms. These findings improve our understanding of within-host dynamics of *S*. Typhi in cases of persistent infection and inform epidemiological investigations of transmission events associated with chronic carriers.

## Introduction

*Salmonella enterica* subsp. *enterica* serovar Typhi (*S*. Typhi) is the major cause of typhoid fever [1]. This disease is contracted via the faecal-oral route and remains a global public health challenge with estimated 10.9 million infections and 116,900 deaths each year world-wide [2]. The disease disproportionately impacts people in developing countries [2, 3]. The emergence and spread of multi-drug and extensively drug resistant *S*. Typhi have further challenged control and treatment of typhoid fever globally [1, 4].

After resolution of clinical symptoms, some individuals become carriers with intermittent shedding of bacteria, with lengths ranging from three weeks (defined as convalescence carriage; three weeks to three months) to greater than a year (chronic carriage) [1]. Persistently shedding carriers not only pose a health risk to others in their vicinity but also contribute as a reservoir for *S*. Typhi circulation, especially in endemic regions [1, 5]. Through animal models (using *S*. Typhimurium instead of *S*. Typhi) and clinical investigations, the key niche in humans for *S*. Typhi carriage has been determined to be the gallbladder [6–9]. This colonisation site exposes *S*. Typhi to bile, a hostile bodily fluid with antimicrobial properties. Mechanisms employed by *S*. Typhi to survive in bile include biofilm formation on gall stones [7, 10, 11], oxidative stress management [12], and sustained cell envelope component synthesis [13]. In addition, Transposon Directed Insertion Site Sequencing also revealed 169 genes that were essential for survival in bile with the most significant genes being the ones required for lipopolysaccharide (LPS) synthesis [14].

Selective pressures during gallbladder carriage could also induce genetic variation in *S*. Typhi, with a study reporting higher numbers of non-synonymous single nucleotide polymorphisms (SNP) detected in *S*. Typhi genomes isolated from the gallbladder when compared to *S*. Typhi recovered from acute patients within the same catchment population [15]. The same study also suggested that non-synonymous mutations were localised to genes responsible for stress response and LPS biosynthesis and that carriage was not necessarily driven by treatment failures due to presence of antimicrobial resistance markers [15]. Another means of intracellular genomic flux within bacteria are mediated by insertion sequences, small genomic segments encoding a transposase, flanked by terminal inverted repeats that are either identical or imperfect [16]. The impact insertion sequences have on bacterial genomes include genomic rearrangements, genomic duplication, deletions, gene inactivation and gene expression changes [17, 18]. In addition, environmental stimuli have been reported to induce transpositions of IS elements, which in turn would further impact the bacterial genome [19–22].

In this study, we interrogated within-host dynamics of genomes of *S*. Typhi recovered from two household cases. The first patient (henceforth referred to as “Case A”) was admitted to a hospital with fever of unknown origin in a country with low prevalence of typhoid. *S*. Typhi was detected in blood culture and the patient was successfully treated for typhoid. As the Case A did not have any history of recent travel to endemic countries all household contacts were screened for *S*. Typhi. *S*. Typhi was recovered from the stool sample of one household contact (henceforth referred to as “Case B”). Case B recalled a previous episode of typhoid fever approximately six years prior in a high incidence country. Sequential isolates of *S*. Typhi were recovered from stool samples collected from Case B across a period of over 142 days and whole genome sequencing (WGS) suggested genetic variation in these isolates with three sub- populations. Metagenomic sequencing of bile fluid obtained during elective surgery confirmed and illuminated features of sub-populations in Case B.

## Materials and methods

### Culture and handling conditions

*S*. Typhi isolates used in this study were cultured from clinical samples at the Centre of Infectious Disease and Microbiology Laboratory Services (CIDMLS), the Institute of Clinical Pathology and Medical Research (ICPMR), NSW Health Pathology. Cultured isolates, grown on XLD plates were forwarded to the New South Wales Enteric Reference Laboratory, ICPMR, for serotyping according to the Kauffmann–White scheme [23]. Isolates were subcultured from the forwarded plate onto a fresh XLD agar prior to DNA extraction and serotyping. All isolates were incubated at 37°C for 18 ± 2 hours. Bile fluid collected for metagenomics was snap frozen after receipt in the laboratory from operating theatre and stored in -80°C until extraction. Prior to extraction, frozen bile samples were placed on ice until fully thawed.

### DNA extraction and sequencing of isolates and bile metagenome

Genomic DNA for whole genome sequencing (WGS) was extracted from five randomly-picked colonies from each subcultured plate using the DNeasy Blood and Tissue kit (Qiagen) following an overnight lysis with Proteinase K (Qiagen). Quality and quantity of extracted DNA was measured spectrometrically (Nano300; AllSheng). For bile metagenomic sequencing, two millilitre of bile fluid was centrifuged for two minutes at 10,000g with supernatant removed and the pellet resuspended with sterile Phosphate Buffered Saline (PBS). This process was repeated twice before resuspending in the pellet in 200 μL of sterile PBS. Host DNA depletion was performed as previously described with the addition of saponin (Sigma-Aldrich) to a final concentration of 0.025% [24]. The saponin treated sample was centrifuged for two minutes at 10,000g after DNase treatment and supernatant was removed. Resulting pellet was resuspended in 800 μL of solution CD1, a component of the QIAamp® PowerFecal® Pro DNA Kit (Qiagen). DNA extraction was subsequently performed using the QIAamp® PowerFecal® Pro DNA Kit according to manufacturer’s instructions. Two millilitres of PBS from same PBS stock were treated the same way throughout the entire extraction procedure as a “kitome” control. DNA quality and quantity was measured spectrometrically (Nano-300; AllSheng) and fluorometrically (Qubit^TM^ 2.0 with dsDNA HS Assay Kit; Invitrogen), respectively. Paired-end 2 x 150 bp libraries were prepared using the Nextera XT library preparation kit (Illumina) according to manufacturer’s instructions and sequenced on the NextSeq 500 (Illumina; 150-cycle mid output) and MiniSeq (Illumina; 150-cycle mid output) platforms for WGS and metagenomics sequencing, respectively.

### Quality control, assembly and annotation for WGS and metagenomics

Illumina reads for both WGS and metagenomics were quality controlled using FastQC version 0.11.3 (https://www.bioinformatics.babraham.ac.uk/projects/fastqc/) and trimmed using Trimmomatic version 0.36 [25]. For both WGS and metagenomic sequencing, taxonomical classification was performed with Centrifuge version 1.0.4-beta [26] against prebuild p+h+v index (12/06/2016 version). Reads classified as either *Homo sapiens* (NCBI taxid: 9606), Human endogenous retrovirus K113 (NCBI taxid: 166122) or synthetic construct (NCBI taxid: 32630) were removed post taxonomical classification using the rextract script packaged with Recentrifuge version 1.9.1 [27]. Residual reads classified as *Homo sapiens* due to multi-classification were removed using the filterbyname.sh script from BBMap version 38.86 (https://sourceforge.net/projects/bbmap/). Taxonomical classifications generated by Centrifuge were visualised on Krona version 2.8 [28]. Unless used within a within a software package, mapping of sequencing reads onto reference genomes was performed using BWA mem version 0.7.17-r1188. Mapping statistics were collated using BAMStats version 1.25 (http://bamstats.sourceforge.net/). *S*. typhi lineage and related antimicrobial resistance markers were determined from the trimmed reads via GenoTyphi version 1.9.1 [29] implemented on Mykrobe version 0.10.0 (https://github.com/Mykrobe-tools/mykrobe).

Trimmed reads from WGS and metagenomics were assembled using SPAdes version 3.14.1 [30]. Assembly of the WGS reads were performed with the “—careful” flag while metagenomic assembly was performed with the “—meta” flag. Contigs less than 300-bp were removed from analysis. When required, annotations were performed using Prokka version 1.14.6 with the flags “—gcode 11” and “—metagenomic”. All genomes were visualised on Artemis version 18.1.0 [31]. Pairwise comparisons were visualised on Artemis Comparison Tool version 18.1.0 [32]. Multiple genome alignment by BLASTN was visualised using BRIG version 0.95-dev.0004 [33]. Scanning for virulence genes was performed on the genomic assembly using Abricate version 1.0.0 (https://github.com/tseemann/abricate) against the Virulence Factor Database [34].

### Core SNP and sub-consensus detection

Variant calling and core SNP alignment was performed using snippy version 4.6.0 (https://github.com/tseemann/snippy) against a *S*. Typhi reference (see results for reference selection) using default settings. When required, pseudomolecules of reference genome with SNPs of their original source genome incorporated were obtained from the snippy output file with the suffix “.consensus.subs.fa”. SNP distance matrix of core SNP alignments were obtained using snp-dist version 0.8.2 (https://github.com/tseemann/snp-dists). Genomes with five or less core SNPs between them were classified as a “cluster”, as per standard operating thresholds for *Salmonella* species utilised by the Microbial Genomics Reference Laboratory, ICPMR [35]. In this study, sub-consensus variants are defined as nucleotide positions showing mixed nucleobases at a single site, with the proportion of reference and alternate nucleobases falling between 10% and 90%. Where required, positions of sub-consensus variants from both WGS and metagenomic sequencing were detected from the mapping files using LoFreq version 2.1.5 [36] on default settings. Distributions of sub-consensus nucleobases in identified positions were quantified using bam-readcount version 1.0.1 [37] and its associated parsing script. Mapping profiles of these positions with sub-consensus variation were also manually inspected on the Integrative Genome Viewer version 2.8.6 [38].

### Genomic inference of IS*10R* insertions

Insertions of IS*10R* were inferred from both an automated and a manual process Automatic inference of insertion site position was performed on ISMapper version 2.0.2 [39] on default settings with the genomic sequence of IS*10R* (Genbank accession: J01829.1; which included sequences for terminal inverted repeats) as a query. Manual inference was performed by initially searching each assembly for the presence of both the terminal inverted repeat upstream of the transposase (IRL) and the terminal inverted repeat downstream of the transposase (IRR) in the assemblies. Insertion of IS*10R* result in target duplication of nine base-pairs (DRs) adjacent to the IRL and IRR [40] and all nine bases adjacent to the 5’ of the identified IRL and IRR sequences were assumed to be its associated DR. The associated DR, along with an additional 11 adjacent bases (to decrease chances of mismatch) was used to search the genome of 129-0238-M (NCBI assembly accession: GCA_001359025.2). Insertion sites were called when both associated DR overlapped and detected on both the positive and negative strand of the reference genome. Once called, the first base of the associated DR in the genome of 129-0238-M was designated its insertion site. Insertion sites were assumed to be identical when the location identified manually, and by ISMapper, is within ± 10 bases of the position identified manually to account for discrepancies when searching using a 9 bp DR sequence from the manual search.

### Inferences of homoplasic sites

A maximum likelihood (ML) tree was reconstructed from the core SNP alignments of the six *S*. Typhi isolates, along with the assemblies of *S*. Typhi strain CT18 [41], *S*. Typhi strain Ty2 [42] and an additional 63 *S*. Typhi lineage 4.3.1.1 assemblies from a previous study in Australia [43, 44], using default heuristic search settings with the GTR+G model in RAxML-NG version 1.2.0 [45]. A *S*. Paratyphi A strain AKU_12601 [46] was used as an outgroup and support for ML tree was assessed with a bootstrap analysis of 1,000 pseudoreplicates. SNPPar version 1.0 [47] was used to identify all possible homoplasic events from both core SNPs and the best ML tree. ML tree were visualised on FigTree version 1.4.4 (https://github.com/rambaut/figtree).

### Ethics statement

Collection of samples were performed under the New South Wales Public Health Act and a non-research determination was granted. Strain names of all isolates used in this manuscript are deidentified.

## Results

### Sequential *S*. Typhi isolates from Case B consisted of three different sub-populations with distinct core SNP patterns

A total of seven isolates, encompassing one from Case A and six from Case B were isolated from multiple clinical specimens across a period of 149 days (Table 1). During this period, Case B did not develop symptomatic disease. When serotyped according to the Kauffmann–White scheme, six isolates possessed an antigenic structure of 9,12,Vi:d:-, representative of *S*. Typhi, while one (CIDM-STyphi-B01) was serotyped as rough, monophasic *Salmonella* (rough,Vi:d-). Assembly statistics of each of the seven genomes are listed in Table 2 while detailed statistics are listed in S1 Table. Assembly of these seven isolates post WGS resulted in genome sizes expected for *S*. Typhi for each of the isolates although CIDM-STyphi-B01 was observed to have an assembly length shorter than the other six genomes (Table 2). At the sequencing and the assembly level, majority (> 97%) of the data were taxonomically classified as *S*. *enterica*. Each assembly possessed an identical 1,392-bp contig that was not taxonomically classified as *S*. *enterica* (Table 1 and S1 Table). BLASTN against the NCBI’s nt/nr database (only inclusive of taxid:28901) revealed hits (100% identity over 100% of query length) to genomes of some *S*. Typhi. Further BLASTN against the ISfinder database [48] revealed that this 1,392 bp sequence was an intact IS*10R*.

**Table 1:** Assembly statistics and genomic typing of *S*. Typhi isolates in this study.

Isolate/Sample ID	Case	Clinical sample	Timeline of isolate recovery^a^	Number of contigs^b^	Assembly length (bases)	Assembly N50	GenoTyphi lineage
CIDM-STyphi-A01	Case A	Blood	D-7	91 (90)	4,720,812	97,881	4.3.1.1
CIDM-Styphi-B01	Case B- Sample 1	Faeces	D+0	93 (92)	4,686,206	110,674	4.3.1.1
CIDM-Styphi-B02	Case B- Sample 2	Faeces	D+15	81 (80)	4,721,781	129,329	4.3.1.1
CIDM-Styphi-B03	Case B- Sample 3	Faeces	D+63	80 (79)	4,722,620	125,234	4.3.1.1
CIDM-Styphi-B04	Case B- Sample 4	Faeces	D+65	80 (79)	4,722,052	143,307	4.3.1.1
CIDM-Styphi-B05	Case B- Sample 5	Bile fluid	D+142	78 (77)	4,723,529	144,688	4.3.1.1
CIDM-Styphi-B06	Case B- Sample 6	Intraoperative gall bladder swab	D+142	75 (74)	4,722,683	153,670	4.3.1.1

^a^Timeline measured in days (designated by the letter ‘D’) relative to the isolation of the first sample from Case B

^b^Number of contigs taxonomically classified as *S*. *enterica* listed in parenthesis

**Table 2:** Discriminatory SNPs and gene products for each of the three sub-populations.

Designation	SNP^a^	Sub-population	Gene product	Substitution type
SNP-01	G129054**A**	2	NarY respiratory nitrate reductase 2 beta chain	Non-synonymous
SNP-02	A389021**G**	3	Ribonuclease E	Synonymous
SNP-03	**G**1442660A	1	Cell invasion protein SipD	Synonymous
SNP-04	C2101913**T**	1	Glucose-1-phosphate adenyltransferase	Non-synonymous
SNP-05	C2187487**T**	3	Cyclic di-GMP phosphodiesterase PdeH	Non-synonymous
SNP-06	C2763160**T**	3	Between tRNA-Leu (CAG) and tRNA-Pro (UGG)	Non applicable
SNP-07	G2972613**A**	1	Hypothetical protein	Non-synonymous
SNP-08	G3203886**A**	2	Hypothetical protein	Non-synonymous
SNP-09	G3228505**A**	1	Carbamate Kinase I	Synonymous
SNP-10	**G**3713127A	1	Lysine decarboxylase	Non-synonymous
SNP-11	C4281972**T**	2	Thiosulfate reductase cytochrome B subunit PhsC	Non-synonymous

^a^Discriminatory nucleobase for sub-population assignment highlighted in bold and underlined

*In silico* typing performed on all seven assemblies revealed that they belong to Sequence Type (ST) 1 of multi locus sequence typing (MLST) and GenoTyphi lineage 4.3.1.1, also known as the H58 clade (Table 1). One hundred and fourteen virulence associated genes were detected in each *S*. Typhi genome (S2 Table). BLASTN coverage over the query sequences of these 114 virulence genes were consistent amongst the seven genomes with the exception of the gene *csgG*, which encoded a gene required for curli fibre biogenesis [49]. In the genomes of CIDM-STyphi-B02, CIDM-STyphi-B05 and CIDM-STyphi-B06, the *csgG* gene each had 99.24% nucleotide identity over 94.60% of the query (VFDB accession: VF0103). This shortfall of 5.4% were all associated with the non-detection of the first 45 bases of the *csgG* gene. The rest of the genomes each had 99.28% nucleotide identity over 100% of the query sequence (VFDB accession: VF0103). Prior to clustering via core SNPs, a reference genome had to be chosen. As the *S*. Typhi lineage for our dataset was known, we compared our sequences against the five long-read *S*. Typhi H58 sequences generated by Wong *et al*. [50]. Of the five genomes, the chromosome of 129-0238-M (NCBI assembly accession: GCA_001359025.2) was chosen as it was the closest genome to our dataset with core SNPs difference ranging from 28 to 31 core SNPS. Coverage and mean read depth across 129-0238-M, from each genome, are listed in S1 Table. The reference genome, along with all isolates recovered were each assessed for possibility of large-scale genomic differences and only CIDM-STyphi-B01 had a region that was missing in relation to every other genome in this study (S1 Fig). As the reference genome was not annotated, we also auto-annotated the genome prior to any downstream analysis.

**Fig 1:**
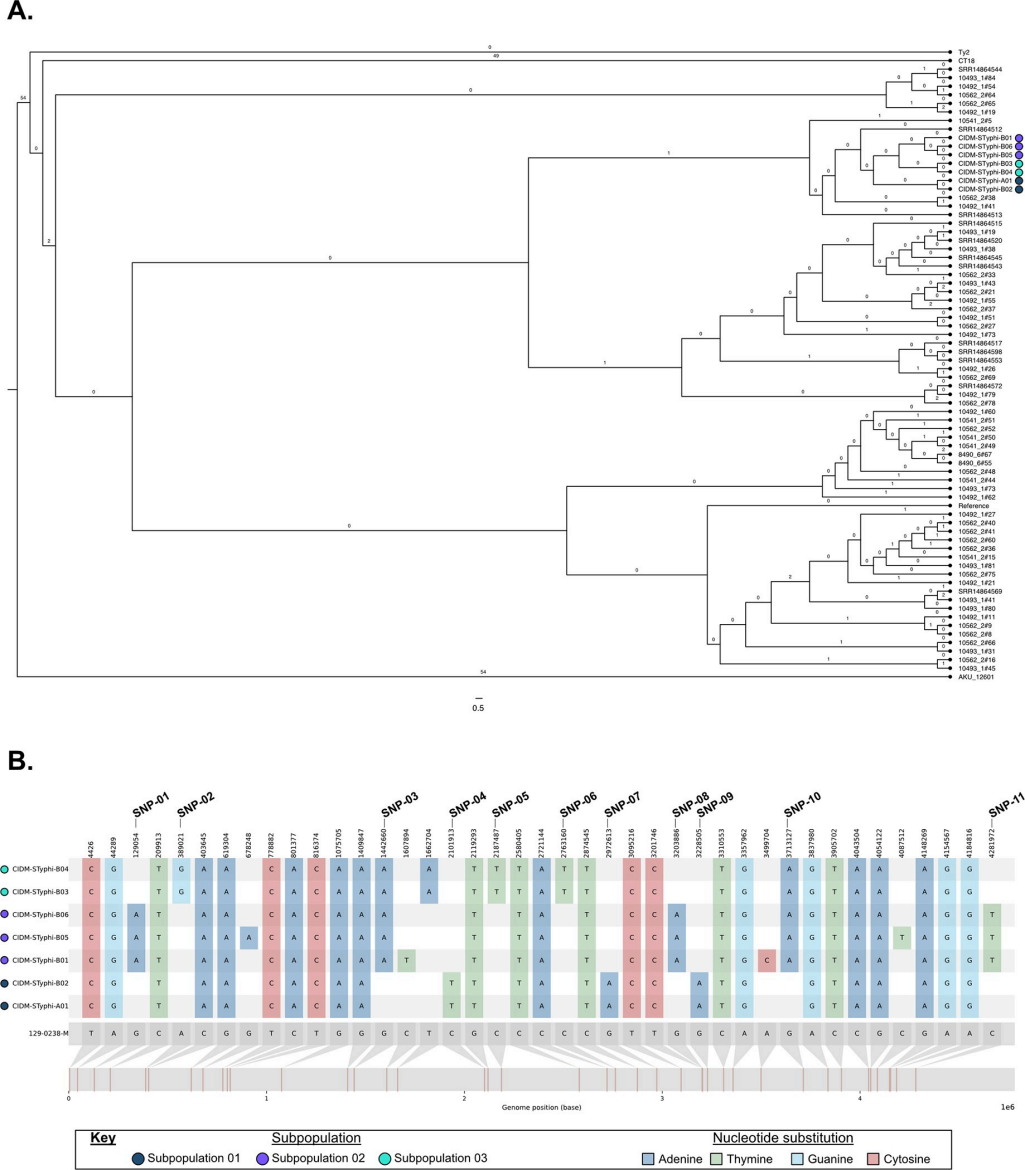
Separation of the seven isolates into three subpopulations. (A) ML tree based on core SNP alignments of the seven isolates along with contextual sequences. Number of detected homoplasic SNPs are labelled on the branches. Number of nucleotide substitutions per site is represented in the scale bar. (B) Comparison of 41 SNPs between the seven *S*. Typhi pseudomolecules and the reference genome. Discriminatory SNP positions are marked in the figure and expanded upon in Table 2. *S*. Typhi populations of the source genomes are represented next to the isolate name and colour-coded according to the Figure Key. Image was generated using snipit (https://github.com/aineniamh/snipit).

Core SNP alignment against the reference genome 129-0238-M, revealed 41 core SNP positions across these seven isolates. Clustering via these core SNPs however, showed some evidence of genetic differences between the seven isolates The isolate from Case A (CIDM-STyphi-A01) and the first isolate from Case B (CIDM-STyphi-B01; collected seven days after Case A’s isolate) did not cluster with each other with the genomes of both isolates separated by nine core SNPs (S3 Table). The second sample from Case B (CIDM-STyphi-B02), collected 15 days after CIDM-STyphi-B01, genomically clustered with CIDM-STyphi-A01 with zero core SNPs differences. CIDM-STyphi-B03 and CIDM-STyphi-B04, isolated approximately two months after CIDM-STyphi-B01, shared zero core SNPs with each other but fell outside the arbitrary threshold of five SNPs for clustering with both CIDM-STyphi-B01 (8 SNP differences) and CIDM-STyphi-A01/B02 (9 SNP differences). CIDM-STyphi-B05 and CIDM-STyphi-B06, isolated from bile fluid and the gall bladder swab, respectively, were obtained during elected cholecystectomy for Case B to treat chronic carriage. Both CIDM-STyphi-B05 and CIDM-STyphi-B06 shared zero SNPs with each other and clustered together with CIDM-STyphi-B01 with one core SNP difference between the three genomes. Based on the clustering of core SNPs, each genome was subsequently clustered into three different sub-population with CIDM-STyphi-A01/B02, CIDM-STyphi-B01/B05/B06 (all of which harboured a truncated *csgG*) and CIDM-STyphi-B03/B04 forming sub-population 01, sub-population 02 and sub-population 03, respectively. Homoplasic SNPs, a marker for adaptive evolution, was not detected amongst the three subpopulations and emergence of the three subpopulations was likely due to vertical inheritance following spontaneous mutations (Fig 1A.)

These 41 SNP were subsequently narrowed down to 11 informative SNP which could be used to differentiate the three sub-populations (Fig 1B). The SNP at position 1,662,704 (Fig 1B) was not considered as it was located within an insertion sequence (IS*200F*) and in both CIDM-STyphi-B03/B04, and there was evidence of mixed nucleotides in this position whereby the sequencing reads were not greater than 95% agreement with each other. At the sequencing read level, all 11 positions showed 100% concordance to the discriminatory nucleobase. Out of these 11 SNPs five, three and three SNPs were discriminatory for sub-population 01, sub-population 02 and sub-population 03 respectively (Fig 1B). Ten informative SNPs were localised in coding sequences with most substitutions being non-synonymous mutations (Table 2).

### Signatures of IS*10R* flanked the missing LPS biosynthesis cluster in the rough *S*. Typhi

**Fig 2:**
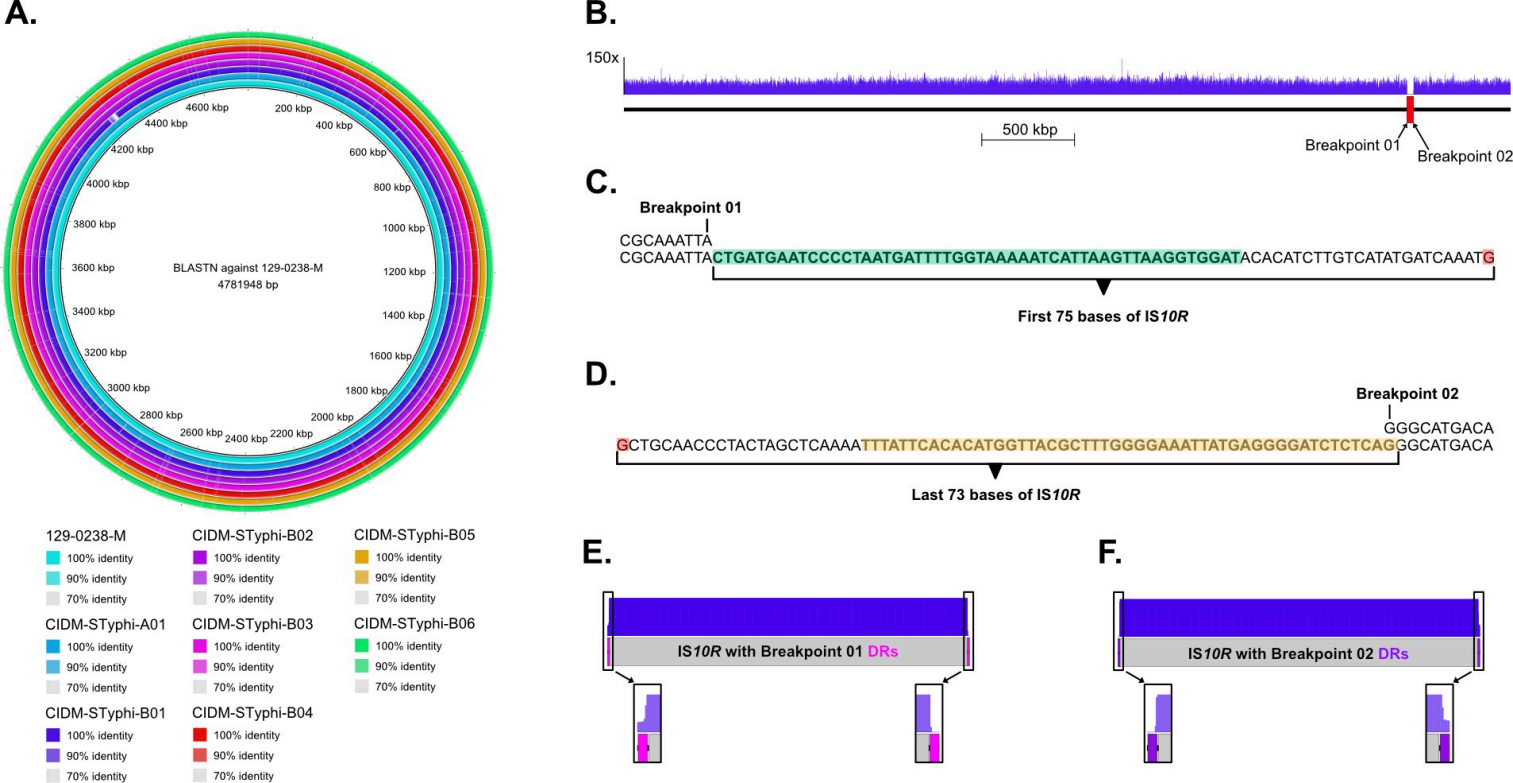
Deletion in CIDM-STyphi-B01 mediated by IS*10R*. (A) The reference genome 129-0238-M is presented in the centre of the ring. Each coloured ring represents a different genome and coloured regions represent BLASTN matches to the reference. Query genomes along with percent BLASTN identity are colour coded in the figure key. (B) Mapping profile of CIDM-STyphi-B01 across the genome of 129-0238-M. Read depths are represented by the blue histogram and the deletion in CIDM-STyphi-B01 is marked up with a red rectangle in its analogous position in 129-0238-M. (C) Assembly of an additional 75 bases in CIDM-STyphi-B01 (bottom) when aligned to breakpoint 01 in 129-0238-M (Top). IRL of IS*10R* highlighted in green. Nucleobase in red represented the end of contig. (D) Assembly of an additional 73 bases in CIDM-STyphi-B01 (bottom) when aligned to breakpoint 02 in 129-0238-M (Top). IRR of IS*10R* highlighted in yellow. Nucleobase in red represented the end of contig. (E) Mapping profile of CIDM-STyphi-B01 across IS*10R* with the expected DR of breakpoint 01 appended. (F) Mapping profile of CIDM-STyphi-B01 across IS*10R* with the expected DR of breakpoint 02 appended. Images in panel (A) was generated using BRIG version 0.95-dev.0004 [33] while (B, E-F) where generated using EasyFig version 2.2.5 [51].

CIDM-STyphi-B01 isolate was a rough mutant of *S*. Typhi and had smaller genome than in other isolates sequenced in this study (Table 1). When all seven assemblies were queried against the reference genome 129-0238-M, a 35,965 bp region was missing in CIDM-STyphi-B01 but was present in all other genomes (Fig 2A). This deletion started from position 4,222,642 in 129-0238-M (breakpoint 01) and ended in position 4,258,606 (breakpoint 02). To ensure that this deletion was not an artefact of low coverage, sequencing reads were mapped onto 129-0238-M which showed zero read coverage across this region (Fig 2B). The genomic region between breakpoint 01 and breakpoint 02 consisted of genes responsible for O antigen biosynthesis which would yield the rough phenotype (S4 Table).

Alignment of CIDM-STyphi-B01 against 129-0238-M also revealed that the analogous positions of breakpoint 01 and breakpoint 02 in CIDM-STyphi-B01 were located in two different contigs. At the analogous position of breakpoint 01 in CIDM-STyphi-B01, an additional 75 bases were assembled before a contig break while the analogous position for breakpoint 02 had 73 additional bases assembled before a contig break (Fig 2C and 2D). BLASTN of these additional bases against the ISfinder database matched them to IS*10R* where the 75 bases in breakpoint 01 matched the first 75 bases (inclusive of the IRL) of IS*10R* while the 73 bases in breakpoint 02 matched the last 73 bases (inclusive of the IRR) of IS*10R* (Fig 2C and 2D). The nine base pairs upstream of breakpoint 01 (5’-CGCAAATTA-3’) and downstream of breakpoint 02 (5’-GGCATGACA-3’), in their respective contigs, were assumed to be the DRs. In addition, the sequences of both DRs were different from each other which suggested that one IS*10R* was located at the breakpoint 01 and a different IS*10R* at breakpoint 02. As only one DRs was detected for each IS*10R* at both breakpoints at the assembly level, the sequencing reads were subsequently interrogated for the presence of the corresponding DR for each IS*10R* insertions at the breakpoints. Mapping over a pseudomolecule consisting of IS*10R* flanked by either the DRs of breakpoint 01 (Fig 2E) or breakpoint 02 (Fig 2F) showed that the missing DRs at each breakpoint did not have any reads mapping over it (Fig 2E and 2F). While the limitations with short read sequencing meant that further interrogation could not be performed, the presence of different IS*10R* signatures at the breakpoints indicated that IS*10R* could have mediated the deletion of the O-antigen biosynthesis cluster in CIDM-STyphi-B01.

### Distribution of IS*10R* in the genomes of the *S*. Typhi isolates

**Fig 3:**
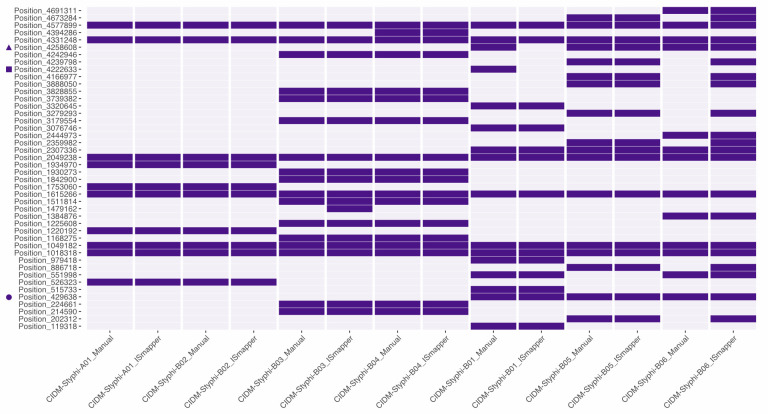
Inference of IS*10R* insertions in each of the seven isolates. Presence and absence matrix of the inferred IS*10R* insertions (nucleobase position relative to 129-0238-M). Purple sectors indicate the presence of a detected IS*10R* insertion site. Detailed information of the insertion site and associated DR sequences are listed in S5 Table. Positions for breakpoint 01, breakpoint 02 and *csgG* insertion are marked with a square, triangle and circle respectively. Figure was generated using ggplot 2 version 3.3.6 [52].

To determine the impact of IS*10R* insertion on other genomic regions, insertion sites of IS*10R* were inferred both manually and via ISMapper. This resulted in a total of 44 IS*10R* insertions were identified across the seven genomes (Fig 3 and S5 Table). Concordance between manual and automated inference of insertion sites were observed in majority of the positions with discordance found in one position in CIDM-STyphi-B03 and eight positions in CIDM-STyphi-B06 (Fig 3 and S5 Table). Another discordance was the two breakpoints that flanked the missing O antigen biosynthesis cluster in CIDM-STyphi-B01 where both insertion sites could be identified manually but could not be detected by ISMapper (Fig 3 and S5 Table). When compared to the other two genomes which constituted subpopulation 02, an IS*10R* insertion site was detected for breakpoint 01 but not for breakpoint02 (Fig 3 and S5 Table).

Inferred IS*10R* insertion sites for CIDM-STyphi-A01 and CIDM-STyphi-B02, which belonged to subpopulation 01, were identical for both but genomes belonging to subpopulation 02 and subpopulation 03 showed some differences within their respective groupings. Majority of the inferred insertions (33/44; 75%) were located within coding sequences and were likely to inactivate the gene it inserted into. One key disruption was the *csgG* gene in genomes belonging to subpopulation 03 (S2 Table), where an IS*10R* insertion was detected within the 5’ end of the gene, which would have resulted in its truncation (Fig 3 and S5 Table).

### Abundance of *S*. *enterica* detected in the bile fluid metagenome

As there were suspicions of sub-population carriage due to the genetic variability of isolates recovered, metagenomics was performed on the bile fluid (CIDM-STyphi-BMG) which was collected at the same time as CIDM-STyphi-B05 and CIDM-STyphi-B06 (Table 1). Metagenomic sequencing generated 12,498,164 and 321 paired reads for CIDM-STyphi-BMG and the kitome control, respectively. In the kitome control two hundred and seven paired reads could not be classified. Majority (n = 71; 62.28%) of the classified reads from the kitome control were taxonomically assigned to *Homo sapiens*, and none of the remaining reads were assigned to *S*. *enterica* (S1 Fig).

**Fig 4:**
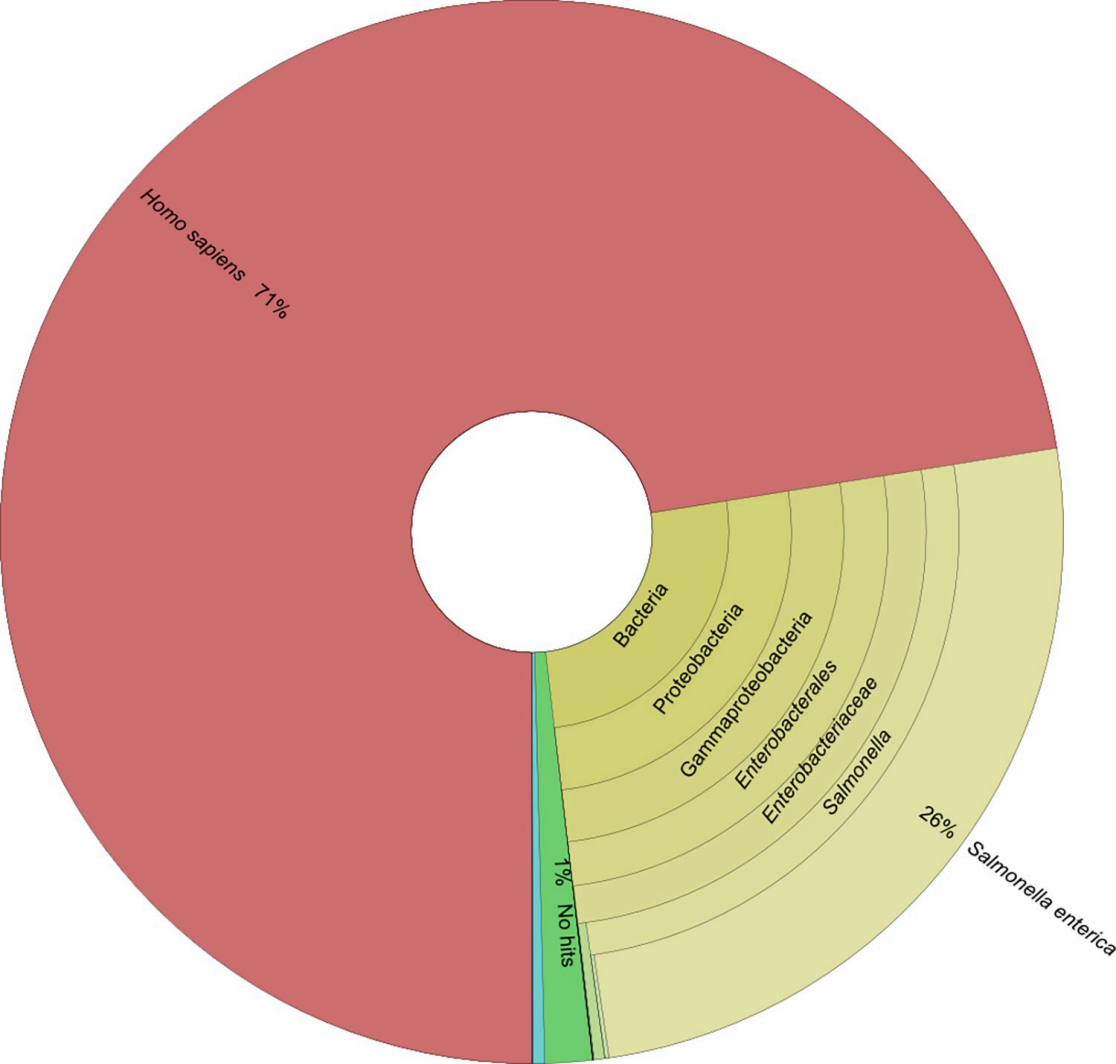
Taxonomical classification of 12,498,164 paired-end metagenomic reads from non-host removed CIDM-STyphi-BMG. Classification was performed against Centrifuge’s prebuild p+h+v index (12/06/2016 version). Image was generated using Krona version 2.8 [28].

Of the 12,498,164 paired-reads generated from CIDM-STyphi-BMG, 189,596 (1.52%) were unclassified and majority of the paired-reads (n = 8,920,640; 71.38%) were taxonomically assigned to *Homo sapiens* (Fig 4). *In-silico* removal of host associated DNA reduced the dataset to 3,342,264 paired-end reads (henceforth referred to as filtered metagenomic reads) where 3,337,531 paired reads (99.86%) were classified as *S*. *enterica*. Assembly of the filtered metagenomic reads resulted in 101 contigs with an assembly length of 4,762,842 bases with an assembly N50 of 89,448 bases. Majority (97/101) of the contigs (> 300 bp) were taxonomically classified as *S*. *enterica* (taxid: 28901). Of the four contigs not classified as *S*. *enterica*, one was the 1,392 bp assembly of IS*10R* while two were classified as *Homo sapiens* (taxid: 9606) and one 308 bp contig was classified as *Simkania negevensis* (taxid: 83561). The latter three contigs that were not classified as *S*. *enterica*, or determined to be IS*10R*, were subsequently removed from further analysis.

### Multiple *S*. Typhi sub-populations in the bile fluid metagenome

The assembled metagenome of CIDM-STyphi-BMG was typed as GenoTyphi lineage 4.3.1.1, similar to the isolates previously recovered from Case B. When the virulome was interrogated, the same set of 114 genes were detected and like CIDM-STyphi-B01, CIDM-STyphi-B05 and CIDM-STyphi-B06, the same disruption in *csgA* was also present (S2 Table). Three of the eleven informative positions, namely SNP-01, SNP-08 and SNP-11, were assembled to their respectively discriminatory SNPs for sub-population 02 which included the isolates CIDM-STyphi-B01, CIDM-STyphi-B05 and CIDM-STyphi-B06. This finding was not unexpected as the isolates CIDM-STyphi-B05 and CIDM-STyphi-B06 were recovered from fluid and swab obtained during the elective cholecystectomy. While CIDM-STyphi-B01 had missing O antigen biosynthesis genes, CIDM-STyphi-BMG possessed the full complement.

**Fig 5:**
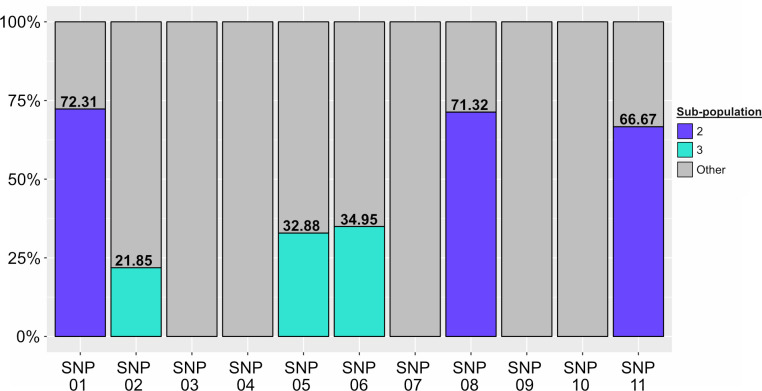
Proportions of the three sub-populations in CIDM-STyphi-BMG inferred from read mapping data over the 11 discriminatory SNP positions. Stacked histograms are colour-coded according to the figure key. As each position is discriminatory for only one sub-population, reads that do not carry the discriminatory SNP for each position were classified as “Other”. Proportions of sub-populations are listed above their coloured bar. Stacked histogram generated using ggplot 2 version 3.3.6 [52].

To determine if there was sub-consensus variation (defined in materials and methods) that would be masked in the assembly level, the filtered metagenomic reads were subsequently mapped onto the genome of 129-0230-M and the discriminatory SNP positions were investigated for sub-consensus within the metagenome. Mapping resulted in a coverage of 99.97% of the chromosome with a mean read depth of 195.45x ± 43.18.991x. At the sequencing read level, discriminatory SNPs for sub-population 02 showed mixed nucleobases at all positions, with the discriminatory nucleobase making up only 70.10 ± 3.01% of the reads (Fig 5). When rest of the nine SNP positions were interrogated, it was determined that the discriminatory SNPs for sub-population 03, namely SNP-02, SNP-05 and SNP-6, also had mixed nucleobases with discriminatory nucleobase making up 29.89 ± 7.04% of the reads. There was no evidence for mixed nucleobase at any discriminatory SNP position for sub-population 01 (Fig 5). In addition to these six positions, mixed nucleobases were also detected at 52 other positions, where the majority were located within repetitive elements (S6 Table). Of these 52 positions, two positions with sub-consensus variants were unique to CIDM-STyphi-BMG (S6 Table), with the first located in an intergenic region (position: 1,386,158) and the second located within the gene which encoded the Autotransporter adhesin SadA (position: 2,276,735; synonymous substitution). Notwithstanding the additional identification of two new positions, the distribution of signatures over six of eleven positions strongly suggested that at the time of sampling the bile metagenome consisted of two sub-populations of *S*. Typhi with sub-population 02 being dominant while sub-population 03 being the minority.

## Discussion

This study utilised a rare set of longitudinal isolates of *S*. Typhi recovered from stool and sterile sites of epidemiologically linked cases, including blood and bile fluid, and interrogated their diversity using genomic and metagenomics approaches. The chronic carriage with diverse *S*. Typhi population was suspected when the first stool isolate from a contact was found to be phenotypically and genomically different from the isolate recovered from Case A with typhoid fever. Subsequent isolates recovered from this individual from different sites, allowed for the clustering of all isolates into three different sub-populations based on core SNPs. In addition, we also reported on 11 discriminatory SNPs that could separate our datasets into the aforementioned sub-populations. These informative SNPs were subsequently used as markers for the quantification of *S*. Typhi in the gallbladder (using bile fluid as a proxy) at the time of elective cholecystectomy. Metagenomic sequencing of the bile fluid revealed that only two sub-populations were accounted for, 142 days after the isolation of the first S. Typhi isolate from Case B’s stool.

Key to separating the different *S*. Typhi isolates into three sub-populations were the identification of discriminatory SNPs in each genome. Apart from splitting the isolates into different sub-populations, this also provided the genomic data to support the epidemiological supposition of house-hold transmission. In non-endemic countries like Australia, chronic carriers of *S*. Typhi, acquired during prior residence in endemic countries, can unknowingly infect household contacts due to intermittent shedding [53, 54]. While the initial stool isolate from Case B was genetically distinct (nine core SNPs) from the isolate associated with bacteraemia in Case A, there was another isolate that was recovered from a separate stool sample that was zero core SNPs apart and they were grouped together as sub-population 01. In addition, both genomes in sub-population 01 showed indistinguishable inferred insertion sites of IS*10R*. The Public Health Unit had investigated for local sources of typhoid for Case A including household stool screening [55]. These results confirmed the epidemiological hypothesis that typhoid fever in Case A was a result of household transmission from Case B. A five SNP-cut off was used to define a genomic cluster but the number of core SNPs amongst all the isolates ranged from zero to nine. In addition, our phylogenetic analysis indicated that the three subpopulations likely descended from a common ancestor. This suggested that a strict SNP threshold cut-off for genomic cluster definition might be overly conservative and does reflect potential diversity of *S*. Typhi sub-populations in the site of persistence within carriage hosts.

In addition to nucleotide polymorphisms, the examination of IS*10R* in the genome appeared to be also informative for epidemiological purposes. Insertion sequences like IS*10R* are the smallest transposable unit in bacteria and intracellular transposition of this unit can have significant impact upon the bacteria chromosome [16]. IS*10R* belongs to the IS4 family, IS10 group for which transposition occurs using a “cut and paste” mechanism [56] The largest impact of IS*10R* had on the genomes in our dataset was the large-scale deletion in CIDM-STyphi-B01, the first isolate recovered from Case B, which led to the O antigen to not be typed serologically with the associated risk of missing typhoid diagnosis. Interestingly, the IS*10R* signatures that flanked the deletion were not entirely intact. The IS*10R* located at breakpoint 01 had a missing IRR associated DR while the IS*10R* located at the breakpoint 02 had a missing IRL associated DR. The other genomes belonging to sub-population 2, CIDM-STyphi-B05 and CIDM-STyphi-B06, also had an inferred IS*10R* insertion at breakpoint 02 but both DRs and the IRL and the IRR were accounted for in these two genomes. These insights suggested that the CIDM-STyphi-B01 evolved from a progenitor sub-population 02 with a IS*10R* being “cut-and-pasted” from a different site in its genome into breakpoint 01. This would generate a genomic organisation whereby the region of deletion is flanked by direct repeats of IS*10R*, each with their own DRs from their insertion site. Subsequently, homologous recombination between these two insertion sequences could produce a single hybrid insertion sequence with DRs from both insertion sequences and the deletion of the genomic region between them [56–60]. However, to fully confirm this hypothesis for the generation of a hybrid IS*10R*, long-read sequencing would be required to provide the genomic context.

This loss of the O-antigen meant that both bile resistance and gallstone biofilm formation in CIDM-STyphi-B01 was negatively impacted [10, 14]. This would also explain why other isolates belonging to subpopulation 02 all had an intact O-antigen and that there were no traces of O-antigen deletions within the metagenome. We had assumed that this deletion event would have occurred serendipitously within the gallbladder of Case B, but we also acknowledge that was also possible that this occurred during efforts to culture the organism from stool samples in the laboratory.

This study has added new insights into the dynamics of *S*. Typhi chronic carriage. A previous study on chronic carriers has suggested that long term carriage of *S*. Typhi in gallbladder would introduce point mutations in the genome due to the exposure to bile, leading to genetic variation [15]. While we focused on longitudinal samples from different sites of epidemiologically linked cases, instead of one isolate per case sampled in a restricted capture area, our findings are complementary. Two of our isolates, CIDM-STyphi-B05 and CIDM-STyphi-B06, which were recovered directly from bile fluid and gallbladder respectively, showed signs of genetic variability. When the SNP profile of CIDM-STyphi-B05, CIDM-STyphi-B06 and CIDM-STyphi-01 were compared, it was not as stratified as the genomes belonging to sub-population 01 and sub-population 03 as there were unique SNPs observed. In addition, when the insertion sites of IS*10R* were inferred, the genomes belonging to sub-population 02 similarly had more occurrences of unique insertion sites compared to the rest of the genome. As they were isolated directly from bile and gallbladder (in contrast to other isolates from stool or blood), these isolates would have been under pressure from bile up to the minute they were plated and thus bile would have been a selection pressure acting upon them which could contribute the genetic variability [15]. In addition, transposition of insertion sequences can be induced by environmental stimuli, especially in events of bacteria stress [19–22] and this could also explain the variability of IS*10R* insertions in genomes belonging to sub-population 02.

Our study findings indicate and quantify the simultaneous carriage of *S*. Typhi sub-populations within the human gallbladder. While our seven isolates showed signs of genetic variability, they were still genetically similar due to the small number of SNPs separating them, an event that likely occurred during bile exposure [15]. Through direct sequencing of the bile fluid, we showed that the persistent infection in Case B was associated with at least two sub-populations of *S*. Typhi, sub-population 02 and sub-population 03, with the former being dominant. We did not detect the signatures for sub-population 01 in the fluid, however, this is not an indication that it was no longer carried at the time of sampling. Only approximately 12.5 million reads were generated in the sequencing run and that possibly with a higher output [61], we might be able to detect signatures of that sub-population. We were fortuitous to have *a priori* knowledge of discriminatory SNPs from the isolates to look out for and thus was able to call a mixed carriage, rather than only a dominant sub-population. This issue is not unique to our application but in all culture independent and targeted sequencing, especially if SNPs are the only discriminatory markers [62]. While having *a priori* knowledge of the SNPs was vital in our study to determine sub-population carriage, the limited number of isolates meant that we could have likely missed additional sub-populations. A case in point was the identification of two additional positions with sub-consensus variation in the metagenome, which were not present in the sequencing data of the seven isolates. While we are unable to determine if these two positions could be markers for a different sub-population, its presence added additional evidence supporting the claim of multiple sub-populations within the chronic carrier.

As metagenomics was only performed on one time point, it is difficult to assess dynamics of *S*. Typhi population, leading to the dominance of one sub-population within the bile fluid. However, the observation of *csgG* being disrupted by IS*10R*, in the genomes of CIDM-STyphi-B01, CIDM-STyphi-B05, CIDM-STyphi-B06 and the metagenome assembly of CIDM-STyphi-BMG could be a contributing factor. The *csgG* gene is part of the two-component operon that encode for curli fibres [49]. In Enterobacteriaceae, curli fibers are a major constituent in the extracellular matrix (ECM) and have roles in adhesion and the establishment of biofilms [10, 63, 64]. The product encoded by *csgG* forms a secretion channel which exports the curlin subunits CsgA and CsgB into the extracellular environment and disruptions of *csgG* have been previously shown to prevent the assembly of curli fibres [65]. The gallbladder is a permissive niche for chronic carriage where *S*. Typhi form biofilms on cholesterol rich gallstones [7, 10, 11]. *In vitro* studies have shown that disruption to curli significantly affected the ability of biofilms [64] and this disruption in members of sub-population 2 would mean that their ability to form biofilms would be compromised and the cells would more likely exist in a planktonic state within the bile fluid. As the bile fluid had no visible gallstones, this could account for the higher proportion of sub-population 02.

In conclusion, our findings based on high-resolution genomic and metagenomics sequencing highlighted the within-host diversity of *S*. Typhi populations in chronic carriers. The detection and investigation of insertion sequences IS*10R* and associated deletions can extend the genome similarity assessments traditionally limited to single nucleotide polymorphisms. These findings improve our understanding of within-host dynamics of *S*. typhi in cases of persistent infection and inform epidemiological investigations of transmission events associated with chronic carriers. Future genomic study of typhoid carriage should consider metagenomic approach or sequencing of multiple randomly selected colonies from a single sample. Chronic carriage is a secondary, albeit a long-lasting facet of *S*. Typhi infections, and better understanding of genomic variability and bile resistance as well as population dynamics are important for the eradication of this host-limited high-burden pathogen.

## Supporting information

S1 Table Antigenic structure, sequencing, and assembly statistics of the seven isolates and the metagenome used in this study. (XLSX)

S2 Table Coverage across virulence genes detected in the assemblies of the seven *S*. Typhi isolates when screened against VFDB. (XLSX)

S3 Table SNP distance Matrix of the seven isolates used in this study. (XLSX)

S4 Table Missing genes of the *rfb* cluster in CIDM-Styphi-B01 relative to genomic locations in 129-0238-M. (XLSX)

S5 Table Inferred insertion site of IS*10R* based on analogous positions in 129-0238-M. (XLSX)

S6 Table Sub-consensus variation from CIDM-STyphi-BMG identified from mapping of reads onto 129-0238-M and the detection of variation in the sequencing data of the seven isolates. (XLSX)

S1 Fig Multiple genomic comparison of the genomes in this study. The reference genome, as demarcated in the figure, is presented in the centre of the ring. Each coloured rings represents a different query genome and coloured regions represent BLASTN matches to the reference. Query genomes along with percent BLASTN identity are colour coded in the figure key. The outermost ring in alternating red and blue represent the contigs of the reference genome, separated by black lines. Image was generated using BRIG version 0.95-dev.0004.
(TIF)

S2 Fig Proportion of the classified bacteria reads from the kitome. Classification was performed against Centrifuge’s prebuild p+h+v index (12/06/2016 version). Image was generated using Krona version 2.8.
(TIF)
