# Supplementary figures and images for: Persistent Salmonella enterica serovar Typhi sub-populations within host interrogated by whole genome sequencing and metagenomics
Source: PLoS One. 2023 Aug 23;18(8):e0289070. doi: 10.1371/journal.pone.0289070 (PMC10446203; doi:10.1371/journal.pone.0289070)

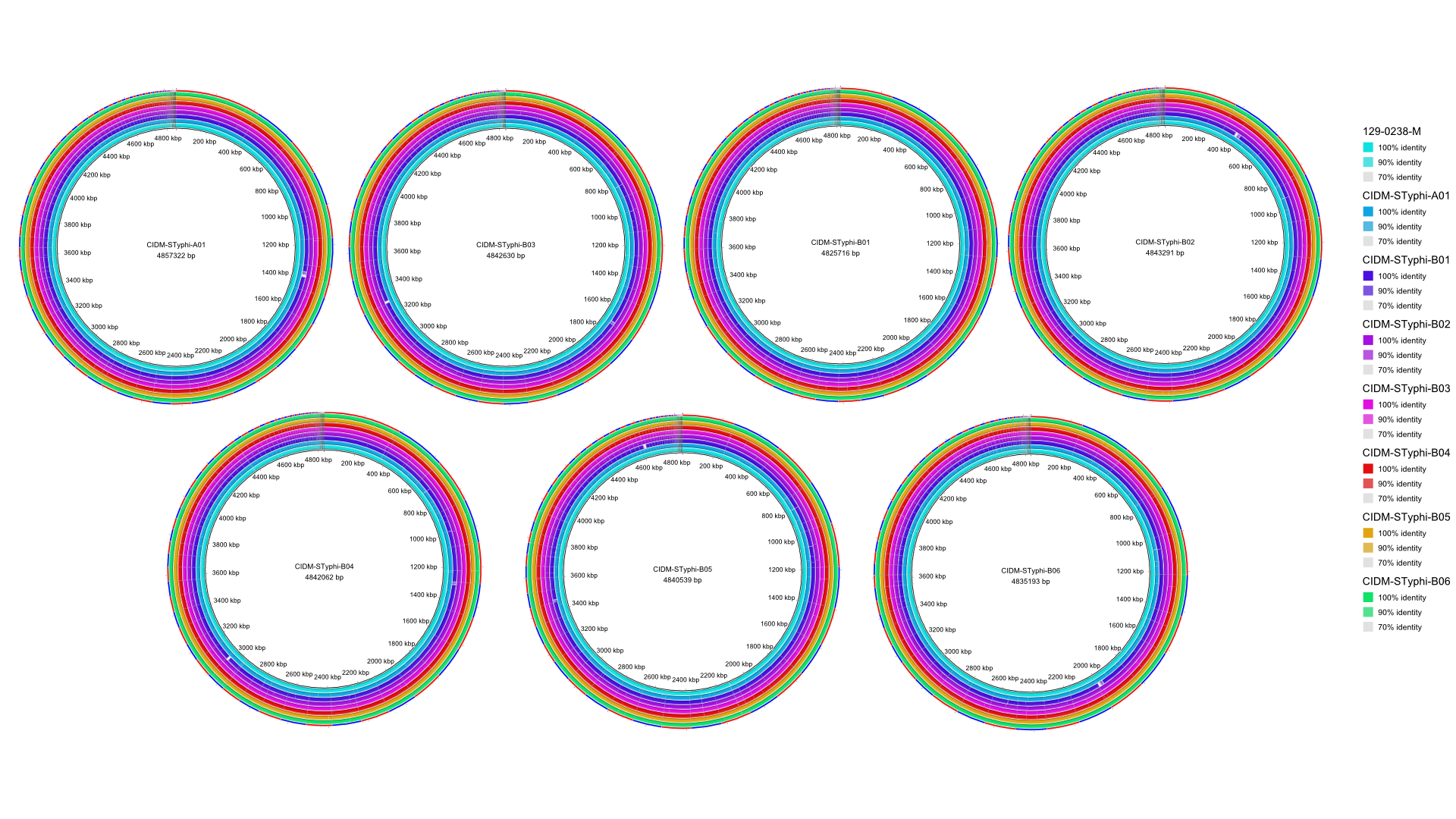

Supplement: S1 Fig — The reference genome, as demarcated in the figure, is presented in the centre of the ring. Each coloured rings represents a different query genome and coloured regions represent BLASTN matches to the reference. Query genomes along with percent BLASTN identity are colour coded in the figure key. The outermost ring in alternating red and blue represent the contigs of the reference genome, separated by black lines. Image was generated using BRIG version 0.95-dev.0004. (TIF) [file pone.0289070.s007.tif]

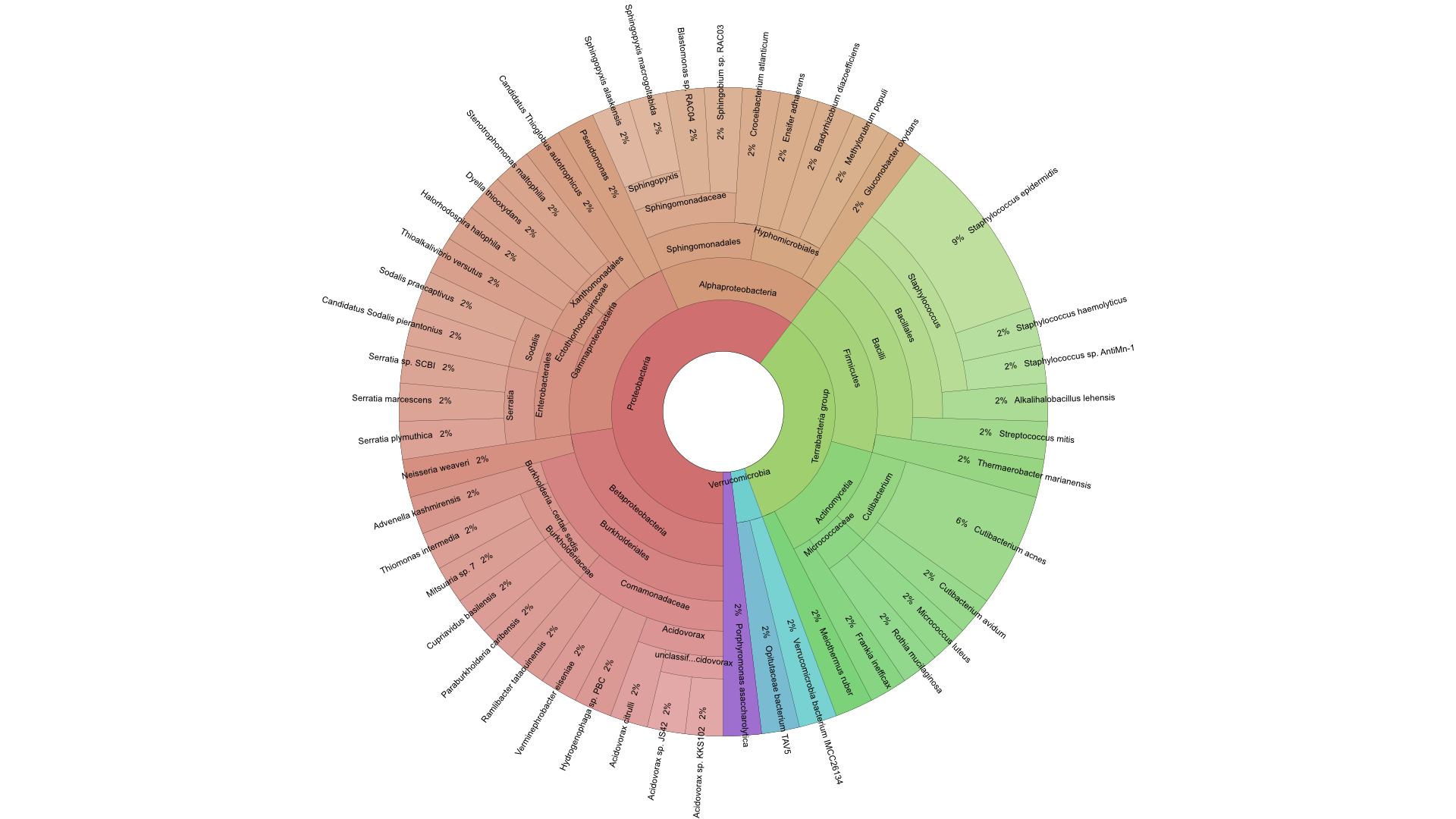

Supplement: S2 Fig — Classification was performed against Centrifuge’s prebuild p+h+v index (12/06/2016 version). Image was generated using Krona version 2.8. (TIF) [file pone.0289070.s008.tif]
